# Efficacy of ivabradine in patients with poor heart rate control after beta-blocker use in acute myocardial infarction: a pragmatic randomized controlled trial

**DOI:** 10.3389/fcvm.2025.1560639

**Published:** 2025-04-03

**Authors:** Yongbin Li, Ying Ren, Lisong Cheng, Xin Zhou, Wenting Li, Lingli Zhang, Jian Cui, Zhuhua Yao

**Affiliations:** Department of Cardiology, Tianjin Union Medical Center, The First Affiliated Hospital of Nankai University, Tianjin, China

**Keywords:** ivabradine, beta-blocker, acute myocardial infarction, heart rate, real-world study, pragmatic randomized controlled trial

## Abstract

**Introduction:**

A rapid resting heart rate following acute myocardial infarction (AMI) predicts poor prognosis, making heart rate control crucial in treatment. Ivabradine is commonly used as a second-line therapy when beta-blockers are poorly tolerated. However, its efficacy in improving cardiac function and prognosis compared to beta-blockers alone remains unclear. This study aimed to investigate the efficacy of ivabradine in the “real world” in patients with AMI who exhibited poor heart rate control despite beta-blocker therapy.

**Methods:**

A total of 1,632 patients with AMI were screened, and 104 patients with resting heart rate >70 bpm after beta-blocker use were randomized in a 1:1 ratio into two groups: the ivabradine (*n* = 52) and control groups (*n* = 52). Metoprolol succinate administration was continued in the control group, whereas the ivabradine group received additional ivabradine administration to achieve a target heart rate <70 bpm. Patients were followed up in outpatient clinics at 3, 6, and 12 months after discharge, during which heart rate, blood pressure, echocardiography, and N-terminal pro-B-type natriuretic peptide (NT-proBNP) were assessed. The primary endpoints were hospitalization for heart failure and cardiovascular death within 12 months. A Cox proportional hazards regression model was used to analyze the risk factors affecting these endpoints.

**Results:**

There were no statistically significant differences in age, sex, risk factors, cardiac function class, blood pressure, heart rate, and comorbid medications between the two groups at the time of enrollment (*P* > 0.05). The ivabradine group achieved significantly lower heart rates compared to the control group at the time of discharge and at 3, 6, and 12 months (*P* < 0.05). At 3 and 6 months after discharge, the Left ventricular ejection fraction (LVEF) in the ivabradine group was higher than in the control group (*P* < 0.05), and the NT-proBNP level was significantly lower than in the control group (*P* < 0.05). Kaplan–Meier survival analysis and log-rank test revealed no statistically significant differences in the incidence of hospitalization for heart failure and cardiovascular death between the two groups at 12 months of follow-up (*P* > 0.05). Cox proportional hazards modeling analysis showed that Killip classification [hazards ratio (HR) = 1.953, 95% confidence interval (CI): 1.207–2.698, *P* = 0.012] and peak NT-proBNP value during hospitalization (HR = 2.096, 95% CI: 1.117–3.075, *P* = 0.028) were influencing factors of hospitalization for heart failure. Age (HR = 1.209, 95% CI: 1.132–1.287, *P* = 0.001), absence of direct percutaneous coronary intervention (HR = 1.095, 95% CI: 1.040–1.149, *P* = 0.001), and LVEF at discharge (HR = 0.902, 95% CI: 0.807–0.996, *P* = 0.041) were influential factors for cardiovascular death. Ivabradine use did not significantly reduce the risk of the primary endpoint events (hospitalization for heart failure HR = 1.420, 95% CI: 0.699–2.878, *P* = 0.332; cardiovascular death HR = 1.025, 95% CI: 0.792–1.257, *P* = 0.836).

**Discussion:**

In “real-world” patients with AMI and poorly controlled heart rate despite titration of beta-blocker dosing, ivabradine was safe and effective in controlling heart rate and improving LVEF early after discharge. However, it had no effect on the 12-month incidence of hospitalization for heart failure and cardiovascular death after discharge.

## 1 Introduction

A rapid resting heart rate following acute myocardial infarction (AMI) is a predictor of poor prognosis (1–3); thus, heart rate control is a critical component of AMI treatment (4, 5). Current guidelines recommend the early initiation of beta-blockers in all patients without contraindications and gradually titrating the dose to achieve optimal heart rate control (6, 7). However, in clinical practice, due to the negative effects of beta-blockers on myocardial contractility and hemodynamics, some patients are unable to tolerate the beta-blocker dose and have poor heart rate control (8).

Ivabradine is a drug that reduces sinus heart rate without significant hemodynamic effects. It is currently approved for patients with chronic systolic heart failure, and AMI is listed as a contraindication to its use (9). However, ivabradine has been studied in several small randomized controlled trials (RCTs) involving patients with AMI, primarily those with STEMI who underwent successful percutaneous coronary intervention (PCI) and demonstrated hemodynamic stability. In these studies, ivabradine was typically administered early post-PCI (10–15). In real-world clinical practice, however, ivabradine use is delayed and often reserved for patients with poorly controlled heart rates or intolerance to beta-blockers (16), including both ST-segment elevation myocardial infarction (STEMI) and non-ST-segment elevation myocardial infarction (NSTEMI) cases, with treatments ranging from emergency PCI to conservative approaches. Furthermore, ivabradine is sometimes initiated before complete hemodynamic stabilization.

Evidently, the design of the previous RCT studies differs greatly from the clinical reality. Additionally, whether ivabradine improves cardiac function and prognosis compared to beta-blockers alone remains unclear. By conducting a pragmatic RCT that is close to the “real world” setting, this study aimed to evaluate the efficacy and safety of ivabradine in improving heart rate control, cardiac function, and clinical outcomes in patients with AMI and poor heart rate control despite beta-blocker therapy.

## 2 Materials and methods

### 2.1 Population screening

This prospective RCT enrolled patients with a first diagnosis of AMI, including acute STEMI and NSTEMI, who were hospitalized in the Department of Cardiology of the Tianjin Union Medical Center between April 2020 and April 2022. Diagnostic criteria were based on the relevant Chinese Medical Association guidelines (17, 18). Inclusion criteria were as follows: (i) beta-blocker therapy titrated to the maximum tolerated dose (defined as the titration of beta-blocker dose after the occurrence of a blood pressure drop to 90/60 mmHg or related deterioration of cardiac function, hemodynamic instability), with a resting heart rate >70 bpm; (ii) resting heart rate >70 bpm in patients in whom the physician deemed the blood pressure was too low or the cardiac function was too poor to tolerate beta-blocker titration; or (iii) hospitalized patients with resting heart rate >70 bpm for whom the physician considered the beta-blocker titration process was too slow. Exclusion criteria included (i) previous history of heart failure; (ii) atrial flutter or atrial fibrillation at the time of admission; (iii) admission blood pressure <90/60 mmHg; (iv) admission heart rate ≤60 bpm or with second-degree or higher atrioventricular block; (v) severe hepatic impairment (Child–Pugh score >9); and (vi) severe renal insufficiency (creatinine clearance <30 ml/min). The study was reviewed and approved by the Ethics Committee of Tianjin Union Medical Center [(2020) CBP (C04)], and all participants provided written informed consent before enrollment.

### 2.2 Interventions

**Figure 1:**
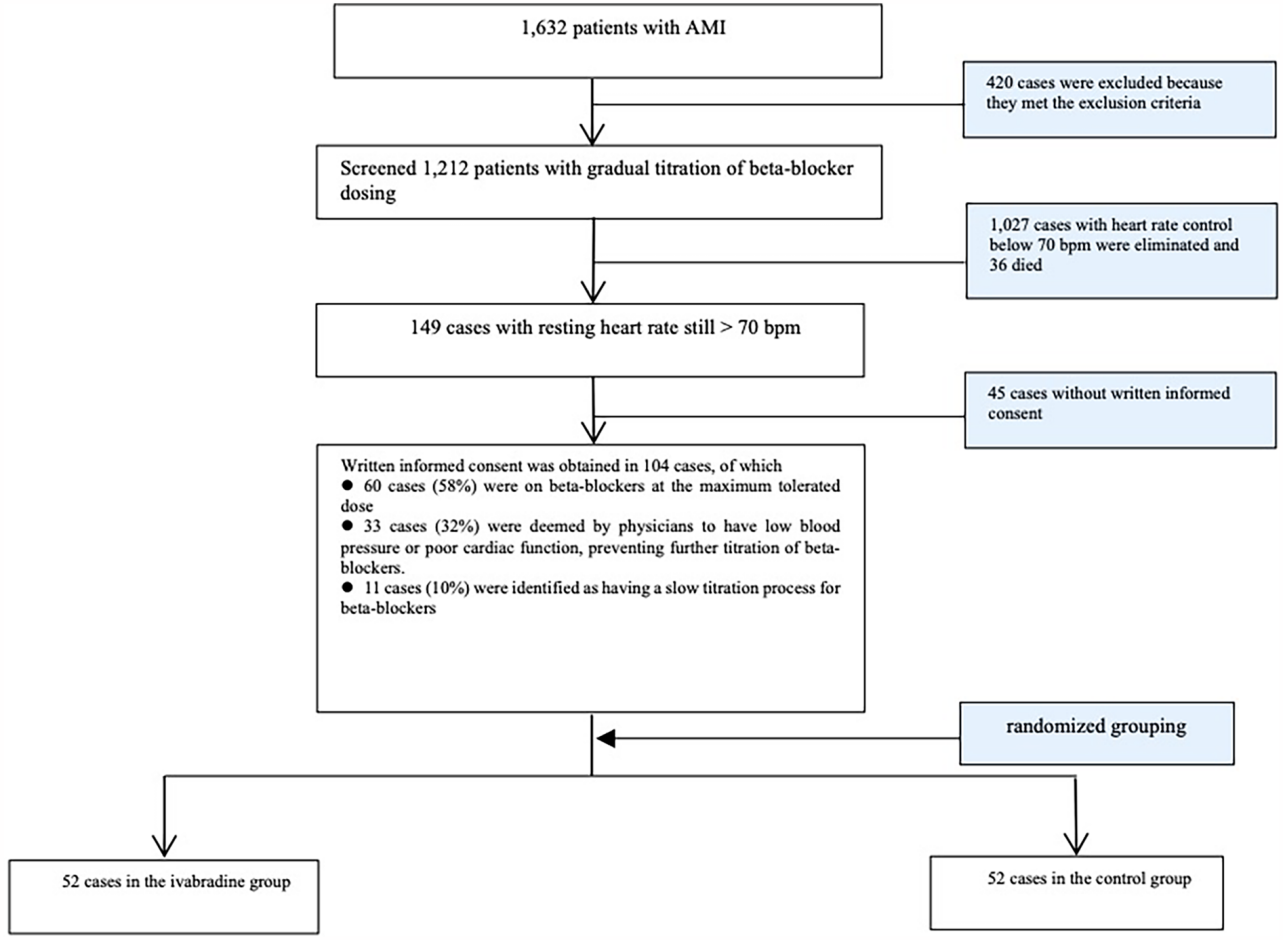
Flow chart for screening study participants.

The screening process was based on a flow chart (Figure 1). All patients with AMI were treated with standardized anticoagulation, antiplatelet, and lipid-lowering therapy according to the guidelines (6, 7), and direct PCI was performed within the treatment time window when clinically indicated. Patients with heart failure were treated to optimize their cardiac function. Those who met the exclusion criteria were excluded according to the flow chart. Eligible patients without contraindications were treated with oral beta-blockers (metoprolol tartrate 25 mg twice daily). After 3 days, the regimen was changed to metoprolol succinate 47.5 mg once daily, with the dose gradually titrated to achieve heart rate control; resting heart rate was measured in the early morning under a quiet condition using a cardiac monitor. Patients who met the inclusion criteria and provided written informed consent were randomized into two groups using a random number table. In the ivabradine group, additional ivabradine hydrochloride tablets (5 mg/tablet; Schweizer, France) were provided to patients already receiving metoprolol succinate, starting at 2.5–5 mg twice daily to achieve a heart rate <70 bpm. In the control group, metoprolol succinate continued to be used to control the heart rate. The ivabradine and metoprolol succinate doses in both groups were adjusted by the clinician based on the patient's heart rate after discharge from the hospital. The following clinical data were collected at the time of enrollment and discharge: general information, type of infarction, Killip classification, LVEF, systolic and diastolic blood pressure, heart rate, whether direct PCI was performed, intra-aortic balloon pump (IABP) implantation, creatine kinase and NT-proBNP peak levels, and comorbid medications, including nesiritide (lyophilized recombinant human brain natriuretic peptide), angiotensin receptor-neprilysin inhibitor (ARNI), and sodium-glucose cotransporter protein 2 (SGLT2) inhibitor. Patients were discharged for outpatient follow-up at 3, 6, and 12 months. During follow-up, resting heart rate and blood pressure were recorded, and echocardiography and NT-proBNP levels were reassessed. The primary endpoints of the study were hospitalization for heart failure and cardiovascular death. Hospitalization for heart failure was defined as an unplanned admission due to worsening symptoms of heart failure (e.g., dyspnea, fatigue, or fluid retention) accompanied by objective evidence of cardiac dysfunction (e.g., elevated NT-proBNP levels, pulmonary congestion on chest x-ray, or reduced LVEF). All heart failure hospitalizations and cardiovascular deaths were confirmed by two independent cardiologists.

### 2.3 Statistical analysis

Statistical analyses were performed using IBM SPSS Statistics for Windows, version 25.0 (IBM Corp., Armonk, NY, USA). Continuous variables following a normal distribution are expressed as mean ± standard deviation $\left(\bar{x}\pm s\right)$, and comparisons between groups were conducted using the independent samples *t* test. Non-normally distributed data are expressed as median (interquartile range) [*M* (Q1, Q3)], and the Mann–Whitney *U* rank-sum test was used for comparisons between groups. Categorical data are expressed as percentages, and the *χ*^2^ test was used for comparison between groups. Survival curve was calculated using the Kaplan–Meier survival analysis, and the log-rank test was used to compare differences in the incidence of primary endpoint events between the groups. The Cox proportional hazards regression model was used to analyze the effects of ivabradine addition, age, Killip classification, cardiac function and other influencing factors on the primary endpoint events. All tests were two-sided, with *P* < 0.05 considered statistically significant.

## 3 Results

### 3.1 Comparison of baseline data between the two groups

The screening process for study participants is illustrated in Figure 1. A total of 1,632 patients with AMI were screened, and 104 patients were ultimately included in the study. The mean age of the participants was 67.1 ± 10.3 years, with 63 (60.6%) being males, 82 (78.8%) diagnosed with STEMI, and 15 (14.4%) having cardiac function class III–IV. A total of 72 (69.2%) patients underwent direct PCI. The average heart rate at enrollment was 85.0 ± 6.5 bpm. Participants were randomly allocated to the ivabradine group (*n* = 52) or the control group (*n* = 52), and no statistically significant differences were observed in baseline characteristics between the two groups (*P* > 0.05) (Table 1).

**Table 1:** Comparison of baseline clinical data between the two groups at enrollment.

Items	Ivabradine group (52 cases)	Control group (52 cases)	Statistical value	*P*-value
Age (year), mean ± SD	68.0 ± 10.3	66.3 ± 10.3	*t* = −0.837	0.405
Male sex, *n* (%)	32 (61.5)	31 (59.6)	*χ*^2^ = 0.040	0.841
Diabetes mellitus, *n* (%)	24 (46.2)	23 (44.2)	*χ*^2^ = 0.039	0.844
Hypertension, *n* (%)	26 (50.0)	27 (51.9)	*χ*^2^ = 0.038	0.309
Hyperlipidemia, *n* (%)	8 (15.4)	9 (17.3)	*χ*^2^ = 0.070	0.791
Smoking, *n* (%)	32 (61.5)	27 (51.9)	*χ*^2^ = 0.979	0.322
Anterior wall myocardial infarction, *n* (%)	29 (55.8)	31 (59.6)	*χ*^2^ = 0.158	0.691
Killip classification (III–IV), *n* (%)	6 (11.5)	9 (17.3)	*χ*^2^ = 0.701	0.402
Direct PCI, *n* (%)	35 (67.3)	37 (71.2)	*χ*^2^ = 0.181	0.671
IABP implantation, *n* (%)	5 (9.6)	7 (13.5)	*χ*^2^ = 0.377	0.539
LVEF (%), mean ± SD	41.4 ± 5.5	41.3 ± 4.7	*t* = −0.077	0.939
CK peak (U/L), M (Q1, Q3)	2,005.0 (483, 4,310)	2,637.5 (483, 4,373)	*Z* = −0.361	0.718
Peak NT-proBNP (pg/ml), mean ± SD	3,006.0 ± 459.7	3,041.7 ± 463.0	*t* = 0.359	0.694
Heart rate at enrollment (bpm), mean ± SD	84.6 ± 6.4	85.4 ± 6.6	*t* = 0.575	0.566
Systolic blood pressure (mmHg), mean ± SD	117.1 ± 21.1	117.3 ± 20.0	*t* = 0.043	0.966
Diastolic blood pressure (mmHg), mean ± SD	72.0 ± 12.6	70.6 ± 12.7	*t* = −0.549	0.584
Nesiritide, *n* (%)	13 (25.0)	18 (34.6)	*χ*^2^ = 1.149	0.284
Intravenous diuretics, *n* (%)	32 (61.5)	29 (55.8)	*χ*^2^ = 0.357	0.550
ARNI, *n* (%)	45 (86.5)	46 (88.5)	*χ*^2^ = 0.088	0.767
SGLT2 inhibitor, *n* (%)	20 (38.5)	22 (42.3)	*χ*^2^ = 0.160	0.689

ARNI, angiotensin receptor neprilysin inhibitor; CK, creatine kinase; IABP, intra-aortic balloon pump; LVEF, left ventricular ejection fraction; NT-proBNP, N-terminal pro-brain natriuretic peptide; PCI, percutaneous coronary intervention; SD, standard deviation; SGLT2, sodium-glucose cotransporter protein 2 inhibitor.

### 3.2 Comparison of changes in heart rate, blood pressure, LVEF, and NT-proBNP levels between the two groups

Both groups showed a significant decrease in heart rate at discharge compared to that at enrollment. The ivabradine group exhibited a significantly lower heart rate compared to the control group at discharge and during follow-ups at 3, 6, and 12 months (*P* < 0.05). There was no statistically significant difference in systolic and diastolic blood pressure between the two groups. Similarly, no significant difference was observed in LVEF and NT-proBNP levels between the two groups at the time of discharge (*P* > 0.05). At the 3- and 6-month follow-ups, LVEF was higher, and NT-proBNP levels were lower in the ivabradine group than in the control group, with statistically significant differences (*P* < 0.05). By the 12-month follow-up, there were no significant differences in the LVEF and NT-proBNP levels between the two groups (*P* > 0.05) (Table 2).

**Table 2:** Changes in heart rate, blood pressure, LVEF, and NT-proBNP between the two groups at follow-up.

Time point		HR (bpm)	SBP (mmHg)	DBP (mmHg)	LVEF (%)	NT-proBNP (pg/ml)
At the time of discharge	Ivabradine group (51 cases)	65.3 ± 3.5	110.3 ± 11.6	65.1 ± 7.2	45.0 (42.0, 48.0)	1,329.8 ± 341.8
	Control group (50 cases)	75.7 ± 8.2	110.7 ± 11.8	64.8 ± 7.2	45.0 (42.0, 46.0)	1,312.9 ± 176.6
	*t*/*Z*	−8.305	−0.192	0.667	1.371	0.312
	*P*-value	0.001	0.848	0.868	0.170	0.756
3 months after discharge	Ivabradine group (49 cases)	62.9 ± 5.3	114.6 ± 10.1	65.1 ± 6.4	46.0 (43.5, 48.0)	858.8 ± 216.1
	Control group (48 cases)	74.7 ± 7.5	115.6 ± 8.2	65.5 ± 6.5	44.0 (41.3, 45.8)	987.5 ± 220.9
	*t*/*Z*	−9.036	−0.551	0.319	2.771	−2.899
	*P*-value	0.001	0.583	0.751	0.006	0.005
6 months after discharge	Ivabradine group (43 cases)	63.3 ± 5.3	111.6 ± 11.7	62.9 ± 8.3	47.0 (45.0, 50.0)	594.0 ± 223.6
	Control group (43 cases)	72.7 ± 7.2	113.5 ± 9.8	64.0 ± 7.7	44.0 (41.0, 46.0)	694.2 ± 216.9
	*t*/*Z*	−6.908	−0.830	−0.647	3.325	−2.109
	*P*-value	0.001	0.409	0.520	0.001	0.038
12 months after discharge	Ivabradine group (41 cases)	63.3 ± 4.7	110.6 ± 10.9	64.0 ± 7.9	46.7 ± 4.9	636.9.9 ± 177.8
	Control group (39 cases)	72.8 ± 6.6	115.1 ± 8.3	65.4 ± 7.2	45.5 ± 5.5	624.1 ± 178.4
	*t*/*Z*	−7.369	−2.052	−0.862	1.098	0.322
	*P*-value	0.001	0.043	0.390	0.272	0.748

DBP, diastolic blood pressure; HR, heart rate; LVEF, left ventricular ejection fraction; NT-proBNP, N-terminal pro B-type natriuretic peptide; SBP, systolic blood pressure.

### 3.3 Analysis of drug dose adjustments and primary endpoint events in both groups at follow-up

**Figure 2:**
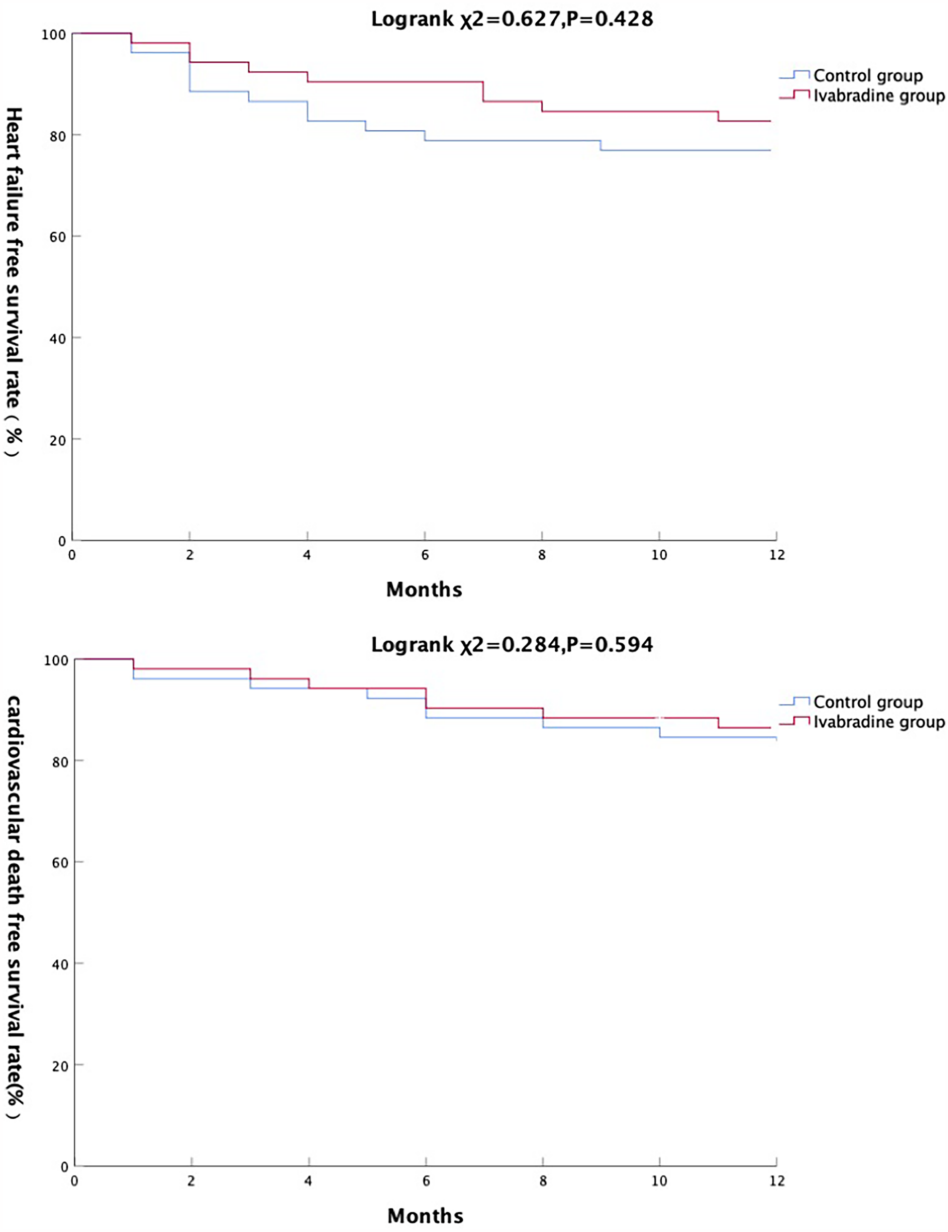
Comparison of primary endpoint events between the two groups.

The ivabradine group was discharged on ivabradine at a mean dose of 6.9 mg/day and metoprolol succinate at a mean dose of 33.7 mg/day. The control group was discharged on metoprolol succinate at a mean dose of 32.6 mg/day. All patients completed 12 months of follow-up. Ivabradine was discontinued in 15 patients from the ivabradine group, Among these, 11 patients were discontinued due to physicians' concerns about bradycardia, and 4 were discontinued due to issues with medication accessibility. Conversely, in the control group, 7 patients were additionally prescribed ivabradine because their heart rates remained above 70 bpm despite reaching the maximum tolerated dose of beta-blockers. At 12 months, the mean doses were as follows: ivabradine group, 7.4 mg/day of ivabradine and 40.5 mg/day of metoprolol succinate; control group, 42.9 mg/day of metoprolol succinate. During follow-up, heart failure occurred in 9 patients (17.3%) in the ivabradine group and 12 (23.1%) in the control group; cardiovascular death occurred in 7 (13.5%) patients (caridiac rupture *n* = 1, recurrent myocardial infarction *n* = 2, heart failure exacerbation *n* = 2, sudden death *n* = 2) in the ivabradine group and 9 (17.3%) patients (cardiac rupture *n* = 2, recurrent myocardial infarction *n* = 2, heart failure exacerbation *n* = 3, sudden death *n* = 2) in the control group. There were no statistically significant differences in the Kaplan–Meier survival curves for heart failure and cardiovascular mortality between the two groups (log-rank test, *P* > 0.05) (Figure 2). During the study period, atrial fibrillation episodes were recorded in 4 patients in the ivabradine group and 5 in the control group, with no statistically significant difference between the two groups (*P* > 0.05).

### 3.4 Analysis of factors influencing heart failure and cardiovascular death in patients with AMI

As shown in Table 3, multivariable Cox proportional hazards regression analysis identified cardiac function class and peak NT-proBNP levels during hospitalization as significant predictors of heart failure events (*P* < 0.05). Age, absence of direct PCI, and LVEF at discharge were significant predictors of cardiovascular mortality (*P* < 0.05). The addition of ivabradine did not reduce the risk of heart failure or cardiovascular mortality (*P* > 0.05).

**Table 3:** Multivariable analysis for the primary endpoint at 12 months.

Risk factor	Multivariable analysis	
	HR (95% CI)	*P*-value
A. Hospitalization for heart failure		
Age	1.410 (0.945–1.874)	0.084
Anterior wall myocardial infarction	1.005 (0.893–1.117)	0.930
Killip classification	1.953 (1.207–2.698)	0.012
Peak NT-proBNP value	2.096 (1.117–3.075)	0.028
Heart rate at enrollment	1.478 (0.887–2.069)	0.113
Additional use of ivabradine	1.420 (0.699–2.878)	0.332
B. Cardiovascular death		
Age	1.209 (1.132–1.287)	0.001
Absence of direct PCI	1.095 (1.040–1.149)	0.001
LVEF at discharge	0.902 (0.807–0.996)	0.041
Combined diabetes	1.053 (0.952–1.153)	0.306
Additional use of ivabradine	1.025 (0.792–1.257)	0.836

CI, confidence interval; HR, hazards ratio; NT-proBNP, N-terminal pro-brain natriuretic peptide; LVEF, left ventricular ejection fraction; PCI, percutaneous coronary intervention.

## 4 Discussion

The present pragmatic RCT was designed to mirror “real-world” clinical setting to assess the efficacy of ivabradine in patients with AMI. Our findings demonstrated that ivabradine was safe and effective in controlling heart rate and improving LVEF early after discharge. However, it had no effect on the 12-month incidence of hospitalization for heart failure and cardiovascular death after discharge.

For patients with AMI and comorbid cardiac insufficiency, the target heart rate remains unclear. This study used 70 bpm as the cutoff point for poor heart rate control, a threshold based on previous studies (19–22) and closely aligned with clinical practice. Among the screened patients, 9.1% still had a heart rate exceeding 70 bpm after using beta-blockers. Notably, 57.7% of these patients had acute anterior wall myocardial infarction, and the mean LVEF was 41.4%, indicating that ivabradine is mainly used in clinical settings for patients with anterior wall myocardial infarction complicated by heart failure. Furthermore, the mean heart rates at discharge were 65.3 and 75.7 bpm in the ivabradine and control groups, respectively, and the heart rate advantage in the ivabradine group persisted throughout the 12 months of follow-up. At 3 and 6 months after discharge, the LVEF was higher and the NT-proBNP level was lower in the ivabradine group compared to the control group, suggesting that patients who had better control of their heart rate with ivabradine also had more significant improvement in cardiac function at the early stage of discharge. Similar findings have been reported in previous meta-analyses (23, 24). The mechanism may be related to the fact that ivabradine improves myocardial perfusion, promotes microangiogenesis, reduces reperfusion injury, and improves left ventricular remodeling (25–27). We also found no difference in the incidence of heart failure and all-cause death at 12 months between the ivabradine and control groups, and the addition of ivabradine did not reduce the risk of the primary endpoint event. However, the separation of the heart failure event curves between the two groups at 2–6 months after discharge suggests that for patients with AMI with heart failure, there may also be a “vulnerable period” of heart failure at the early stage of hospital discharge. Ivabradine appeared to provide a protective effect in this period; however, there was no difference in the incidence of heart failure between the two groups at 12 months, and the addition of ivabradine did not reduce the risk of the endpoint event.

Increased heart rate in patients with AMI is strongly associated with poor long-term cardiovascular prognosis (19). However, it remains unclear whether heart rate itself can be an independent therapeutic target (28). The long-term benefit of beta-blocker use in patients with AMI with preserved ejection fraction (>50%) has also been recently questioned (29). In the present study, all participants had AMI with reduced ejection fraction <50%), and the results showed that the addition of ivabradine to the maximally tolerated dose of beta-blockers for heart rate control did not confer additional benefit. There are two possible reasons for this outcome: First, the increase in heart rate in these patients may be a compensatory response, and solely controlling the heart rate will not only weaken this compensatory mechanism but also mask the nature of the heart failure. Second, for patients with AMI and comorbid heart failure, physicians tend to add drugs such as ARNI and SGLT2 inhibitors during the hospitalization period; the widespread use of these drugs may have diminished the effect of ivabradine. Notably, patients with AMI with reduced ejection fraction represent a population at high risk of progressing to chronic heart failure with reduced ejection fraction (30, 31). The significance of heart rate control in these patients is even greater (32), and ivabradine may hold important therapeutic value during the stabilization period of treatment.

This study has certain limitations. First, as a pragmatic RCT, there may be inherent biases in the inclusion criteria and intervention measures. Second, the availability of several drugs for the treatment of heart failure may have diluted the role of ivabradine in preventing the progression of heart failure. Finally, due to the pragmatic design of this study, there was a crossover of patients between the two groups during follow-up, which may have influenced the findings of the study.

In conclusion, this study demonstrates that in “real-world” patients with AMI who have poorly controlled heart rate despite titration of beta-blocker dosing, ivabradine is safe and effective in controlling heart rate and improving left ventricular function in the early discharge period but has no effect on the incidence of hospitalization for heart failure and cardiovascular death at 12 months after discharge. Future multi-center studies with larger sample sizes and longer follow-up periods are warranted to confirm our findings.

## Data Availability

The raw data supporting the conclusions of this article will be made available by the authors, without undue reservation.
